# Assessment of biocompatibility for citric acid crosslinked starch elastomeric films in cell culture applications

**DOI:** 10.1038/s41598-025-90933-0

**Published:** 2025-02-21

**Authors:** N Pooja, Nafisa Yeshmin Ahmed, Sib Sankar Mal, Prasad A. S Bharath, Guan-Yu Zhuo, Hemanth Noothalapati, Vishwanath Managuli, Nirmal Mazumder

**Affiliations:** 1 Department of Biophysics, Manipal School of Life Sciences, Manipal Academy of Higher Education, Manipal, 576104 Karnataka India; 2 Department of Chemistry, National Institute of Technology, Suratkal, 575025 Karnataka India; 3 Department of Public Health Genomics, Manipal School of Life Sciences, Manipal Academy of Higher Education, Manipal, 576104 Karnataka India; 4 Institute of Biophotonics, National Yang Ming Chiao Tung University, Taipei, 11221 Taiwan; 5 Department of Biomedical Engineering, Chennai Institute of Technology, Chennai, 600069 Tamil Nadu India; 6 Department of Chemical Engineering, Indian Institute of Technology Hyderabad, Kandi, Sangareddy, 502285 Telangana India; 7 Faculty of Life and Environmental Sciences, Shimane University, 1060 Nishikawatsu-Cho, Matsue, 690- 8504 Japan; 8 Department of Mechanical and Manufacturing Engineering, Manipal Institute of Technology, Manipal Academy of Higher Education, Manipal, 576104 Karnataka India

**Keywords:** Thermoplastic starch, Citric acid, Silicon dioxide, Thermoplastic elastomer, Biophysics, Environmental sciences, Materials science

## Abstract

This study investigates the synthesis of potato starch elastomers reinforced with silicon dioxide (SiO_2_) and citric acid as a crosslinking agent to enhance their mechanical and barrier properties. Surface morphology analysis using optical microscopy revealed that pure potato starch films had uneven surfaces. However, higher SiO_2_ concentrations increased roughness, while citric acid crosslinked films displayed smoother surfaces overall. Water vapor transmission rates (WVTR) indicated that native starch films were highly hydrophilic, while SiO_2_ incorporation and citric acid crosslinking significantly reduced WVTR of 17% (30% lower than native film), enhancing the barrier properties. Tensile strength testing revealed that citric acid crosslinking increased the tensile strength by 25%, while SiO_2_ further reinforced the films but decreased elasticity by 15%. SiO_2_ had little impact on degradation rates, while citric acid crosslinking delayed microbial growth, extending film longevity by 20%. Biocompatibility assays using SiHa, HT-29, and HEK 293 cell lines revealed that the films had varying degrees of cell confluency. Films with both SiO_2_ and citric acid showed improved confluency (20% higher) compared to films containing only SiO_2_. However, citric acid alone resulted in the highest confluency (95% viability), suggesting its significant role in biocompatibility. This eco-friendly approach demonstrates substantial advancements in film properties, offering potential applications in diverse biomedical industries.

**Supplementary Information:**

The online version contains supplementary material available at 10.1038/s41598-025-90933-0.

## Introduction

The advancement of tissue engineering and regenerative medicine has driven the need for biocompatible materials that can effectively support cell growth and differentiation. Conventional cell culture substrates made from synthetic plastics have been the mainstay of cell culture practices for decades. Typically used in the form of flat, rigid surfaces like tissue culture plates, flasks, and petri dishes, these materials offer several advantages that have contributed to their widespread use. Traditionally, synthetic polymers such as polystyrene (PS), polyvinyl chloride (PVC), and polyethylene terephthalate (PET) have been the standard choice for fabricating cell culture substrates^1–3^. These materials are favored due to their ease of production, mechanical robustness, and capacity to support cell adhesion. Synthetic polymers such as polystyrene are favored for their optical transparency, ease of sterilization, and mechanical strength, which facilitate the handling and imaging of cultured cells^4^. These attributes have made synthetic polymers the gold standard for cell culture applications. However, despite their widespread use, these materials present several limitations that challenge their continued application in advanced biomedical research and tissue engineering. One of the most significant drawbacks of conventional plastic substrates is their non-biodegradable nature. Derived from petroleum-based sources, these synthetic plastics do not naturally degrade, leading to a buildup of non-degradable materials, especially in long-term in vivo studies or implantable devices^5^. This accumulation presents environmental and health concerns, as it can contribute to chronic inflammation and foreign body reactions when used in medical applications. Furthermore, the disposal of these materials after cell culture experiments contributes to the increasing volume of non-biodegradable waste in the environment, exacerbating the global plastic waste problem^6^. Another limitation of synthetic polymers lies in their inability to closely mimic the natural extracellular matrix (ECM) in terms of both biochemical and mechanical properties. The ECM is a complex, dynamic structure composed of proteins, glycoproteins, and polysaccharides that provide structural support and regulate various cellular functions. Conventional plastics, being rigid and chemically inert, fail to replicate the mechanical softness and bioactivity of the ECM, which can influence cellular behavior. For instance, cells cultured on rigid plastic surfaces often exhibit altered morphology, proliferation rates, and differentiation patterns compared to those in vivo. Additionally, the surface chemistry of synthetic polymers often requires modification with ECM proteins such as collagen or fibronectin to promote cell attachment and proliferation, adding complexity and cost to the cell culture process^7^.

To address these limitations, researchers have explored various biodegradable materials that are more environmentally friendly and biocompatible. These materials are derived from natural sources and are designed to degrade into non-toxic byproducts over time, thereby reducing environmental impact and enhancing their suitability for both in vitro and in vivo applications. Several biodegradable polymers have been investigated for cell culture applications, including polylactic acid (PLA), polycaprolactone (PCL), and naturally derived biopolymers like gelatin, alginate, and chitosan. PLA is a biodegradable and bioresorbable polymer derived from renewable resources such as corn starch and sugarcane. It can be fabricated into various forms, including films, scaffolds, and microspheres, making it versatile for cell culture and tissue engineering^8^. However, PLA’s relatively hydrophobic nature and lack of inherent cell-adhesive properties necessitate surface modifications or blending with other materials to enhance its biocompatibility^9^. PCL, another biodegradable polyester, has a slower degradation rate than PLA, making it suitable for long-term cell culture and tissue engineering applications^10,11^. PCL’s mechanical flexibility and biocompatibility have made it a material of interest for the development of scaffolds and films for cell culture^12^. However, like PLA, PCL is hydrophobic and lacks inherent cell-adhesive properties, necessitating surface modifications or the incorporation of bioactive molecules to promote cell attachment and proliferation^13^. Gelatin, a denatured form of collagen, provides a biomimetic environment that closely resembles the ECM, promoting cell adhesion and proliferation^14^. Alginate, derived from brown seaweed, forms hydrogels that can encapsulate cells, allowing for 3D cell culture and tissue engineering^15^. Chitosan, obtained from chitin, has antimicrobial properties and can be used to fabricate films and scaffolds with tunable mechanical properties^16^. Despite their biological relevance, these materials often lack the mechanical strength required for certain applications and may require crosslinking or blending with other polymers to enhance their properties^17^.

Starch is a naturally occurring polysaccharide that has recently emerged as a promising material for biodegradable films and coatings due to its abundance, low cost, and biodegradability^18–20^. Starch-based materials have been extensively explored in applications such as food packaging, drug delivery, and biopolymer coatings due to their ability to form flexible and biodegradable films^21–24^. Consequently, these composites tailored for packaging applications often lack the biocompatibility, tunable degradation rates, and mechanical properties necessary for effective cell culture applications.

In contrast, our approach aims to leverage the benefits of starch-based materials while tailoring them specifically for biomedical applications, particularly in cell culture. The novelty of our work lies in the development of potato starch-based elastomer films reinforced with silicon dioxide (SiO₂) and crosslinked with citric acid to improve their mechanical and barrier properties. Unlike conventional starch-SiO₂ composites, our elastomer films are designed to provide a biocompatible microenvironment that supports cell adhesion, proliferation, and viability. The incorporation of SiO₂ nanoparticles into the starch matrix enhances the film’s mechanical strength, thermal stability, and resistance to degradation, making it suitable for long-term cell culture applications. Unlike food packaging applications, where SiO₂ primarily serves as a reinforcement agent, our formulation strategically employs SiO₂ to modulate surface properties, hydrophilicity, and cellular interactions. Citric acid, a natural crosslinking agent, further improves the film’s mechanical properties and biodegradability, allowing for the fabrication of films with tunable properties.

Our study aims to assess the biocompatibility of these potato starch-based elastomer films using various cell lines, including SiHa, HT-29, and HEK 293. By evaluating cell adhesion, proliferation, and viability on these films, we aim to determine their suitability as an alternative to conventional plastic substrates for cell culture. This research represents a significant step forward in the development of environmentally sustainable and biocompatible cell culture platforms, bridging the gap between traditional starch-based biodegradable films and next-generation biomaterials for tissue engineering and regenerative medicine.

## Materials and methods

### Materials

Potato starch and Citric acid anhydrous were procured from Loba Chemie Pvt.Ltd, India. SiO_2_, acetic acid and iodine solution were acquired from HiMedia Laboratories Pvt. Ltd, India. Glycerol (extra pure AR, 99.5%) used as a plasticizer was acquired from Sisco Research Laboratories Pvt. Ltd., India. 6 well cell culture plates were procured from Greiner bio-one CELLSTAR. Laminar air flow hood, inverted microscope, micropipettes, water bath set to 37 °C, Dulbecco’s Modified Eagle Medium (DMEM) with 10% FBS, 70% Ethanol, and Trypsin (0.25%) were used for successful culture and passaging of cells. The Neubauer Chamber along with coverslips and trypan blue were used for counting the cells.

## Methods

### Determination of apparent amylose content by amylose binding method

The apparent amylose content (AAC) of native potato starch, used as a raw material for biopolymer synthesis, was determined using the amylose binding assay^25^. In this method, 0.1 g of potato starch was treated with 95% ethanol and 1 N NaOH in a 1:9 ratio. The solution was incubated in a boiling water bath for about 10 min and then cooled to room temperature. The total volume was adjusted to 100 mL with distilled water. To 5 mL of this solution, 1 mL of acetic acid and 2 mL of iodine solution were added, followed by a 20-minute incubation at room temperature (28–30 °C). The absorbance of the blue-colored complex was measured at 620 nm using a UV–Visible spectrophotometer (Varioskan Flash, Thermo Fisher Scientific, USA).

### Synthesis of starch films

The potato starch-based elastomers were synthesized using the solution casting technique^25,26^. 8 g of potato starch and different quantities of SiO_2_ (0.08 g, 0.16 g) and citric acid (50%, 30%, 10%, 5% w/w of potato starch) were dissolved in distilled water to synthesize various batches of biopolymer films. Glycerol at 20% (w/w) of starch was added as a plasticizer during the synthesis process. The beaker was then placed on a hot plate magnetic stirrer (C-MAG HS 7) and heated at 85–90 °C for about 45 min with continuous stirring. The starch gel was carefully poured onto a casting tray, preventing the formation of air bubbles, and allowed to dry in a hot air oven for 48 h. After drying, the biopolymer film was peeled from the casting tray and kept in a dry, humidity-controlled chamber (37 °C and humidity > 50%) until further use. The detail elastomer composition can be found in Table 1.

**Table 1:** Elastomer Composition and codes.

Elastomer name	Potato Starch	Glycerol	SiO₂	Citric Acid
**NPS**	8%	20% (w/w of starch)	-	-
**NPS/0.08 S**			0.08 g	-
**NPS/0.16 S**			0.16 g	-
**NPS/5CA**			-	5% (w/w of starch)
**NPS/10CA**			-	10% (w/w of starch)
**NPS/30CA**			-	30% (w/w of starch)
**NPS/50CA**			-	50% (w/w of starch)
**NPS/0.08 S/10CA**			0.08 g	10% (w/w of starch)
**NPS/0.16 S/10CA**			0.16 g	10% (w/w of starch)
**NPS/0.08 S/30CA**			0.08 g	30% (w/w of starch)
**NPS/0.16 S/30CA**			0.16 g	30% (w/w of starch)
**NPS/0.08 S/50CA**			0.08 g	50% (w/w of starch)
**NPS/0.16 S/50CA**			0.16 g	50% (w/w of starch)

### Thickness

The thickness of the synthesized biopolymer films was measured using a digital micrometer (Yuzuki, Tokyo, Japan; 0.01 mm resolution).

### Transparency

Thermo Fisher Scientific’s Varioskan TM LUX multimode microplate reader was used to determine the percentage of transmittance of biopolymer films. Transmittance, which provides an indirect measurement of transparency, is the amount of light (of various wavelengths) that travels through the sample. The absorbance of the films at different wavelengths (300–700 nm) was converted into percentage transmittance using the following equation.

1 $$\:\varvec{\%}\varvec{T}=\varvec{a}\varvec{n}\varvec{t}\varvec{i}\varvec{l}\varvec{o}\varvec{g}(2-\varvec{A}\varvec{b}\varvec{s}\varvec{o}\varvec{r}\varvec{b}\varvec{a}\varvec{n}\varvec{c}\varvec{e})$$

The surface morphology of the starch films was analyzed at 40x magnification using a bright-field (Olympus BX51, Japan) microscope.

### Water solubility

The initial weights (W_i_) of biopolymer films were calculated after they were cut and dried at 105 °C in the hot air oven overnight. After that, the films were continuously stirred for 24 h while submerged in beakers containing 100 mL of distilled water. The filter paper was used to remove the surplus water from the films, and they were then dried for 24 h at 60 °C^27^. The films’ ultimate weights were noted as the final weight ( W_f_). Using the following equation, the percentage of water solubility was computed.

2 $$\:\varvec{W}\varvec{a}\varvec{t}\varvec{e}\varvec{r}\:\varvec{s}\varvec{o}\varvec{l}\varvec{u}\varvec{b}\varvec{i}\varvec{l}\varvec{i}\varvec{t}\varvec{y}\:=\:\frac{{\varvec{W}}_{\varvec{i}}-{\varvec{W}}_{\varvec{f}}}{{\varvec{W}}_{\varvec{i}}}\times\:100\:\:$$

### Moisture content

The moisture content of starch films was determined by drying 2 cm × 2 cm films in the hot air oven at 60 °C for an extended period until a constant weight was obtained. The moisture content was then calculated by the following equation:

3 $$\:\varvec{M}\varvec{o}\varvec{i}\varvec{s}\varvec{t}\varvec{u}\varvec{r}\varvec{e}\:\varvec{c}\varvec{o}\varvec{n}\varvec{t}\varvec{e}\varvec{n}\varvec{t}\:=\:\frac{\varvec{W}\varvec{e}\varvec{i}\varvec{g}\varvec{h}\varvec{t}\:\varvec{o}\varvec{f}\:\varvec{w}\varvec{a}\varvec{t}\varvec{e}\varvec{r}}{\varvec{W}\varvec{e}\varvec{i}\varvec{g}\varvec{h}\varvec{t}\:\varvec{o}\varvec{f}\:\varvec{d}\varvec{r}\varvec{y}\:\varvec{f}\varvec{i}\varvec{l}\varvec{m}}\times\:100$$

### Water vapour transmission rate (WVTR)

The WVTR of the films was measured by cutting circular films with a 5 cm diameter and securely sealing them around the entrance of glass vials containing 10 mL of distilled water. The initial weight of the vials was noted. The setup was placed in a humidity chamber for 24 h with a temperature of 35 °C and a relative humidity of 40%. The vials were taken out and reweighed to determine the water vapor permeated through the films^28^. The following equation can be used to determine WVTR.

4 $$\:\varvec{W}\varvec{V}\varvec{T}\varvec{R}\:=\:\varvec{\varDelta\:}\varvec{W}\:\varvec{A}\varvec{*}\varvec{t}$$

where ΔW is the weight loss in grams, A represents the cross-sectional area and t is time in h.

### Tensile strength

The tensile strength of the starch elastomers was determined using a Universal Testing Machine (UTM). The tests were performed with a load cell of 500 N capacity at a tensile rate of 1 mm/min. The specimens, with dimensions of 90 mm in length and 20 mm in width, were clamped between the grips, ensuring proper alignment to avoid uneven stress distribution. Three specimens of each film were subjected to testing, and the results were averaged to obtain stress (mm) vs. strain (Kn) plot.

### Fourier transform infrared (FTIR) spectroscopy

The chemical characteristics of the elastomer films were analyzed by capturing their infrared spectra using an FTIR equipped with attenuated total reflection (ATR) optics (Bruker Alpha, Germany) within the wavenumber range of 500–4000 cm⁻¹.

### Biodegradation

The soil burial method to assess the biodegradability of the films was adapted from Wang et al.^29^. The films were cut into pieces measuring 3 cm² each and were placed into pots filled with garden soil and buried at a depth of 2 cm for 30 days within a greenhouse environment. The weight of the films was recorded every ten days following burial. Biodegradability was assessed using the following formula:

5 $$\:\varvec{W}\varvec{e}\varvec{i}\varvec{g}\varvec{h}\varvec{t}\:\varvec{l}\varvec{o}\varvec{s}\varvec{s}\:\left(\varvec{\%}\right)\:=\:\left[\right(\varvec{W}\varvec{o}\:-\:\varvec{W})/(\varvec{W}\varvec{o}\left)\right]\:\times\:\:100$$

Where Wo represents the initial weight of the samples and W represents the weight after the test period.

### Time dependent estimation of pH and absorbance properties of starch elastomers in DMEM

The pH of the surrounding medium was monitored using a pH meter at certain time intervals (0, 2, 4, 6, 8, and 24 h) by immersing different types of starch films in DMEM. The major goal of this experiment was to track the pH changes over time and assess how the films responded and remained stable in the DMEM environment. The films might release basic or acidic substances when submerged in the medium, which could alter the pH of the surrounding solution.

### Biocompatibility

DMEM (4.5 g/l D-Glucose, Gibco) supplemented with 10% fetal bovine serum (Gibco) has been used as the culture medium, and different cell lines were employed for evaluating the biocompatibility and viability of the starch-based films^18^. An inverted microscope was used to look for evidence of contamination and deterioration in the tissue culture flask. With the help of a pipette, the culture media was gently removed from the flask. To rinse the cells and get rid of any remaining media, 3–4 mL of PBS (Phosphate-buffered saline) solution was added to the flask. 200 µl of Trypsin which was at 37 °C was added to the T-25 flask and tilted up and down to ensure that the monolayer was completely covered with Trypsin. Trypsin was pipetted out leaving behind a few drops and then incubated in a 5% CO_2_ incubator for 2–3 min. The flask was gently tapped from the sides to thoroughly dislodge the cells. 1mL of the cell suspension was transferred to a 15mL tarson tube and 20 µL of cells were collected for cell counting. The flask was incubated at 37 °C in a 5% CO_2_ incubator.

Starch-based films were placed into the bottom of the wells of a six-well plate after UV sterilization. Cells were then seeded at a density of 50,000 cells/ mL directly into the starch-based films and covered with 4mL of fresh culture medium for the biocompatibility and cytotoxicity assays. Images were obtained to assess biocompatibility following a 3-day culture period. The control was a six-well plate devoid of film. To determine if cells were adherent to the surfaces, the films were removed from the 6-well plate and placed in a new 6-well plate with fresh culture media^30^.

## Results and discussion

### Synthesis of elastomer films from potato starch with different additives

The synthesis is based on the high-temperature (80 °C) acid hydrolysis of starch with citric acid, followed by polymerization and reannealing utilizing glycerol as a plasticizer. The film-forming solution was obtained after the monomeric units were joined to create polymeric structures. The ability of the starch granules to form films is due to their amylose content. The biopolymer film is made less brittle by the addition of a plasticizing agent. Plasticizers reduce these forces, make the film more flexible, and enhance the mobility of the macromolecules, resulting in better gas or water transmission. However, prior studies also highlighted the effectiveness of glycerol as the plasticizing agent for biodegradable polymers, prompting its use in the current study^31^. Starch, citric acid, SiO_2_, and glycerol amounts were optimized, and it was observed that 8 g/ 100 mL of potato starch (20.26% amylose content determined by amylose binding method) could form optimal biopolymer films. The determination of AAC is crucial for understanding the film-forming ability and structural properties of starch-based materials. Amylose, being a linear polysaccharide, contributes significantly to the mechanical strength, film-forming capability, and water resistance of the starch matrix. In cell culture applications, these properties directly influence the substrate’s suitability by ensuring adequate rigidity, flexibility, and stability in an aqueous environment. High amylose content typically results in films with better mechanical strength and lower solubility, which are essential for maintaining the integrity of the cell culture substrate during long-term use. Additionally, amylose plays a role in modulating the hydrophilicity of the films, which can affect cell adhesion and proliferation. Therefore, assessing the amylose content provides valuable insights into the material’s performance and compatibility with cell culture applications. The addition of SiO_2_was found to increase the stiffness of the films, likely due to the formation of a more rigid network structure within the matrix. Silica nanoparticles are known to act as reinforcing agents in polymer matrices by enhancing the intermolecular interactions and improving the material’s resistance to deformation^32^. However, at higher concentrations, SiO_2_may lead to brittleness due to agglomeration or excessive crosslinking, which reduces the overall flexibility of the film. This phenomenon has been reported in similar studies involving nanoparticle reinforcement^33^. The crosslinked films showed improved flexibility and appeared denser, which is indicative of a more tightly packed polymer network. The increased density can contribute to the film’s enhanced resistance to deformation under stress^26^.

### Characterization of the starch films

#### Thickness

**Fig. 1:**
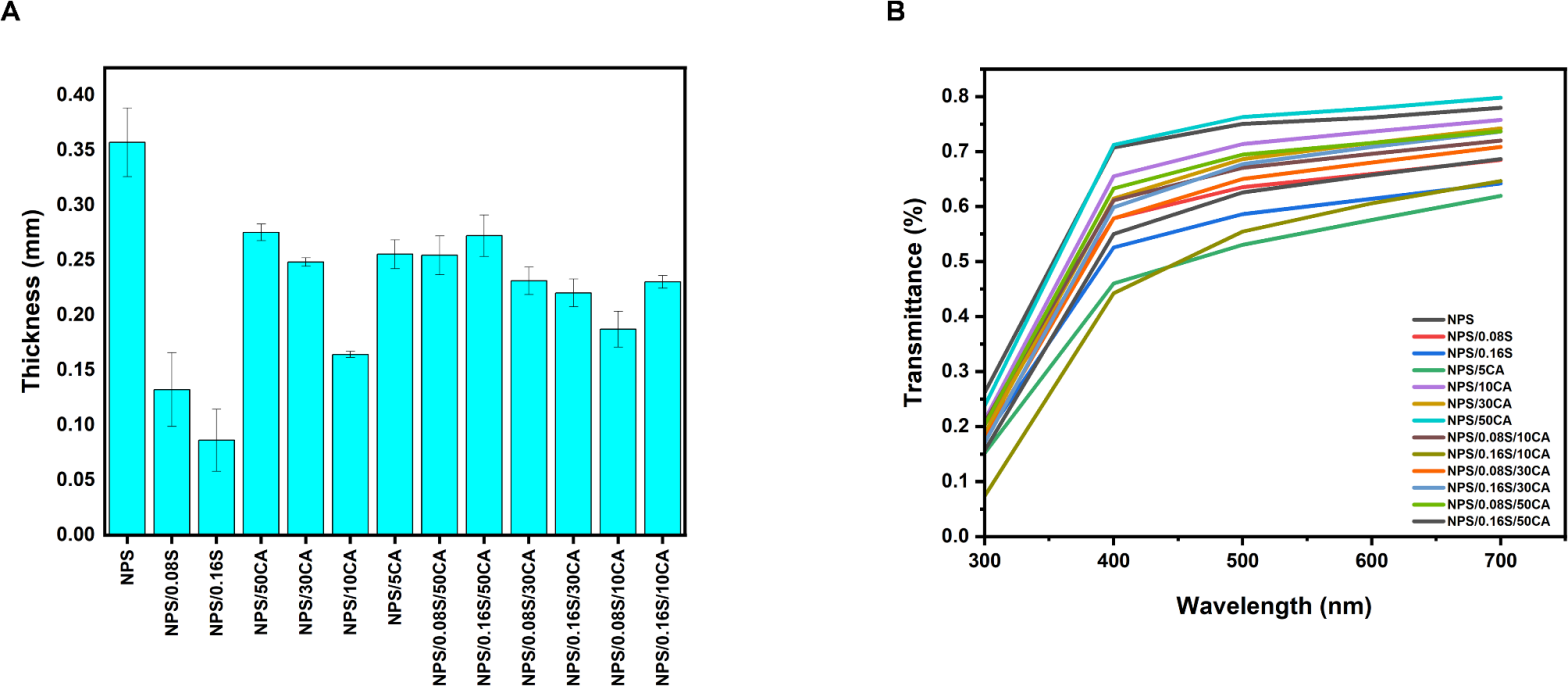
Graphs representing **(A)** thickness; **(B)** transmittance (%) of the synthesized potato starch elastomers.

The thickness of the developed films measured using a digital micrometer is depicted in Fig. 1A. It was observed that the incorporation of SiO_2_reduced the thickness of the films. Reduced thickness could be attributed to the tightness and compactness of the polymeric matrix seen with its addition as a reinforcement filler, as observed in similar studies^34^. The citric acid crosslinked films were found to be visibly thicker since the film thickness is proportional to the total solid content of the formulation. Thicker films provide more structural stability, which is beneficial for prolonged cell culture studies. Crosslinking with citric acid results in a denser polymeric network, reducing degradation rates in aqueous environments which is an important factor for cell culture applications requiring long-term film stability^25^.

#### Transparency

The transparency of films is a critical property for cell culture applications, as it directly affects the ability to monitor cellular behavior using optical microscopy. The percentage transmittance of the films across the wavelength range of 300–700 nm was analyzed and is shown in Fig. 1B. Films crosslinked with citric acid demonstrated enhanced transparency, particularly in the visible spectrum (400–700 nm). This improvement can be attributed to the reduction of voids and the formation of a homogenous polymer matrix through crosslinking^35^. The smoother and denser structure facilitates better light transmission, making these films suitable for cell culture substrates where high optical clarity is essential for visualizing cells during growth and proliferation studies. Previous studies have demonstrated that crosslinked starch with citric acid resulted in enhanced optical clarity^36^. On the other hand, increasing SiO_2_ concentrations resulted in reduced transparency. This reduction is likely due to light scattering caused by the aggregation of SiO_2_within the film matrix^34^. While the reinforcement provided by SiO_2_ improves mechanical properties, the associated decrease in transparency could hinder direct microscopic observation of cells, especially at higher concentrations. The combination of citric acid and SiO_2_ offered a balanced outcome. Films containing both additives maintained sufficient transparency to allow cell observation while also benefiting from improved mechanical and barrier properties. This balance makes these films versatile substrates for cell culture, especially in cases where structural stability is equally important. For ultraviolet light (300–400 nm), all films displayed reduced transmittance, which could offer additional benefits in protecting cells from UV-induced damage during prolonged culture.

#### Water solubility and moisture content

**Fig. 2:**
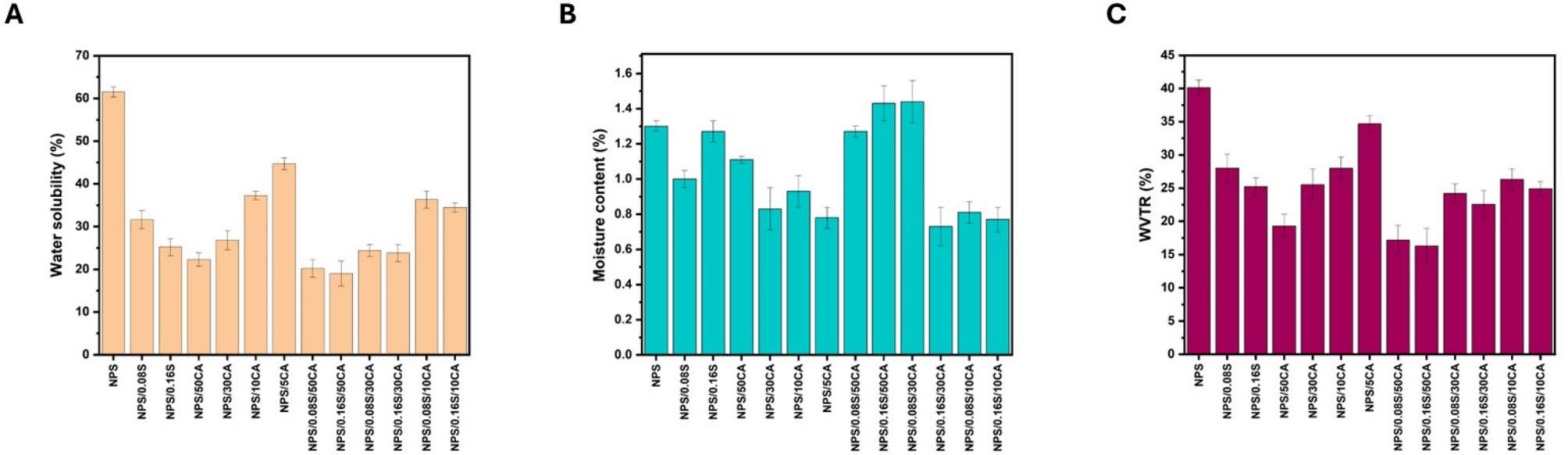
Graphs showing **(A)** water solubility (%), **(B)** moisture content (%), and **(C)** WVTR (%) of the synthesized potato starch elastomers.

Water solubility and moisture content are crucial factors in cell culture applications, as they determine the film’s stability in an aqueous environment. High solubility could lead to premature degradation of the substrate, affecting its ability to support long-term cell growth. The percentage water solubility and moisture content of the bioplastic films are represented in Fig. 2. When compared to potato starch elastomers, the SiO_2_-containing elastomers displayed lower water solubility and moisture content which is in accordance with the literature^32^. The increased water resistance seen in the films might be due to the intricate network created by the intermolecular interactions between starch and SiO_2_ particles. The use of SiO_2_as reinforcement fillers also reduces the overall volume of voids inhabited by water molecules, which lowers the films’ capacity to absorb moisture and water solubility. Citric acid is added to films to further improve their water resistance because it crosslinks with starch molecules to generate strong hydrogen bonds with them^26^. The combination of both citric acid and SiO_2_ films showed improved water solubility and moisture content.

#### WVTR

The WVTR is a critical parameter for films used as substrates in cell culture applications, as it directly impacts the moisture environment surrounding the cultured cells. Maintaining appropriate humidity and moisture levels is essential for cellular health and function.

In the case of native starch films, which are inherently hydrophilic, high WVTR were observed due to their affinity for water. While this hydrophilicity could benefit cellular adhesion and growth by maintaining some level of moisture, the high permeability may lead to excessive water loss, potentially disturbing the moisture balance and affecting cell viability, especially in long-term cultures. The incorporation of SiO_2_ into the starch films provided a marginal improvement in WVTR, as shown in Fig. 2C. SiO_2_particles helped to compact the structure of the film, leading to a denser matrix that slightly reduced WVTR^37^. This improvement may offer a more stable moisture environment, which could be beneficial for cell culture applications where controlling the rate of water loss is crucial for maintaining cellular hydration. Films with high WVTR may lead to excessive evaporation of the culture medium, causing fluctuations in osmotic pressure and negatively affecting cell viability^38^. Citric acid crosslinking resulted in a substantial reduction in WVTR. This reduction can be attributed to the formation of a tighter and more hydrophobic network of starch molecules through crosslinking. The crosslinked films exhibited stronger intermolecular interactions, which limited the free movement of water molecules through the film^39^. For cell culture, this feature is particularly valuable as it helps to create a more controlled microenvironment by preventing excessive dehydration of the cells. By reducing WVTR, the citric acid crosslinked films ensure that moisture levels remain more consistent, supporting the growth of cells, particularly in cultures that require high humidity or long-term maintenance.

#### Tensile strength

**Fig. 3:**
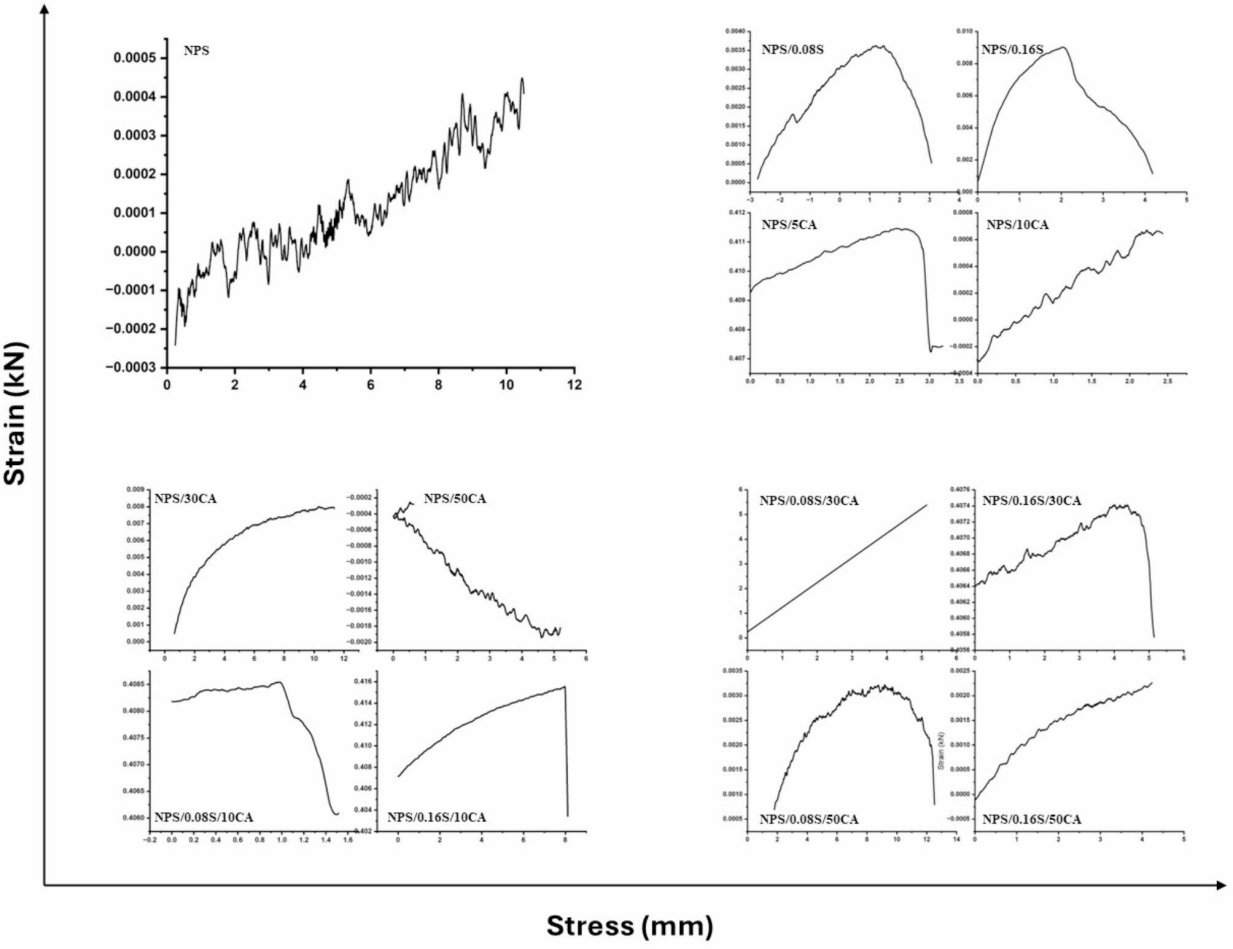
Graph representing stress (mm) vs. strain (kN) plot of the synthesized potato starch elastomers.

The stress vs. strain plot comparing different potato starch elastomers is described in Fig. 3. It was observed that the incorporation of citric acid crosslinking and SiO_2_incorporation had a significant positive effect on the tensile strength of the elastomers. Tensile strength refers to the ability of a material to resist breaking or deformation under tension. In practical cell culture applications, film substrates need sufficient tensile strength to withstand handling during sterilization and cell seeding. The observed increase in tensile strength with SiO₂ reinforcement and citric acid crosslinking suggests that these films can serve as robust cell culture substrates, similar to reinforced hydrogel-based scaffolds used in regenerative medicine^40^. The presence of citric acid as a crosslinking agent likely formed additional chemical bonds between the potato starch molecules, thereby enhancing the overall strength of the elastomer. Furthermore, the addition of SiO_2_was noted to improve the tensile strength of the elastomers^35^. This could be due to the reinforcement of SiO_2_ into the elastomer matrix, increasing its resistance to deformation and improving its strength. However, it was also observed that the incorporation of SiO_2_ resulted in a decrease in elasticity. Elasticity refers to the ability of a material to return to its original shape after being stretched or deformed. The reduction in elasticity could be attributed to the fact that the presence of SiO_2_ may have reduced the available free volume between the biopolymer chains within the elastomer. This reduction in free volume may have limited the flexibility of the elastomer, making it less capable of undergoing large deformations and reducing its overall elasticity. In summary, the observations from the stress vs. strain plot suggest that citric acid crosslinking and the addition of SiO_2_ had significant effects on the tensile strength and elasticity of the potato starch elastomers. The crosslinking improved tensile strength, while SiO_2_ reinforcement increased strength but decreased elasticity, possibly due to the reduction of free volume and flexibility in the elastomer structure. In comparison, a study on boric acid-modified thermoplastic starch films also shows an improvement in tensile strength due to crosslinking, with boric acid forming additional bonds within the starch matrix. However, similar to the findings with SiO_2_, the addition of excess boric acid in TPS films reduces flexibility, indicating a reduction in the material’s elasticity^41^. In both cases, crosslinking agents like citric acid and boric acid improve the mechanical strength of the films or elastomers but can compromise flexibility when used in higher concentrations.

#### FTIR

**Fig. 4:**
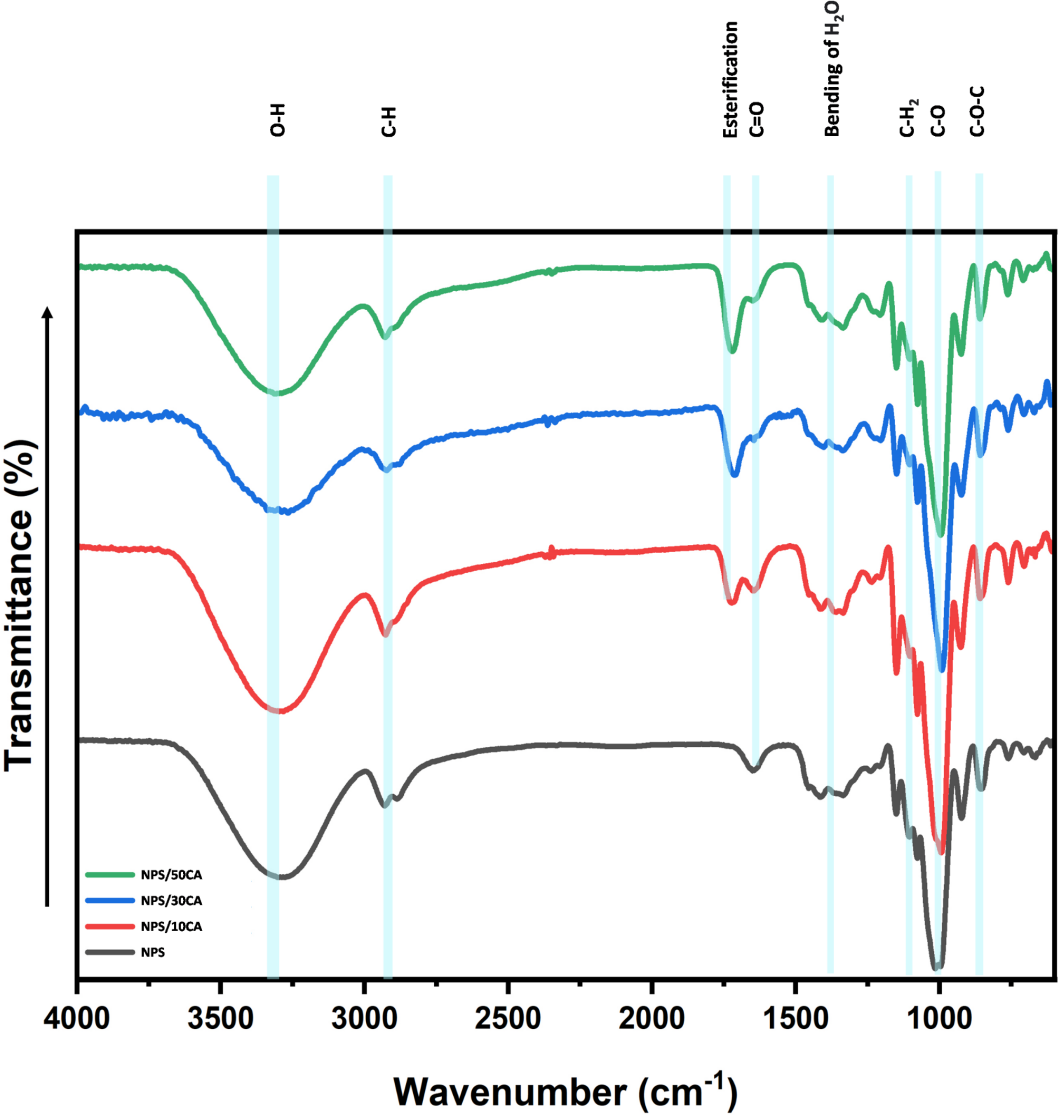
FTIR spectra of synthesized elastomers.

The FTIR spectrum (Fig. 4) provides direct evidence of the chemical interactions involved in the crosslinking process. Notably, the presence of peaks at approximately 1735 cm⁻¹ (C = O stretching of ester bonds) and 1220 cm⁻¹ (C–O stretching) confirms the formation of ester linkages between citric acid and starch molecules during high-temperature crosslinking. Additionally, the broad O–H stretching band around 3200–3600 cm⁻¹ shows a reduction in intensity with increasing citric acid content, indicating reduced free hydroxyl groups due to crosslinking. These observations align with the enhanced mechanical and elastomeric properties observed in the films.

#### Biodegradation

**Fig. 5:**
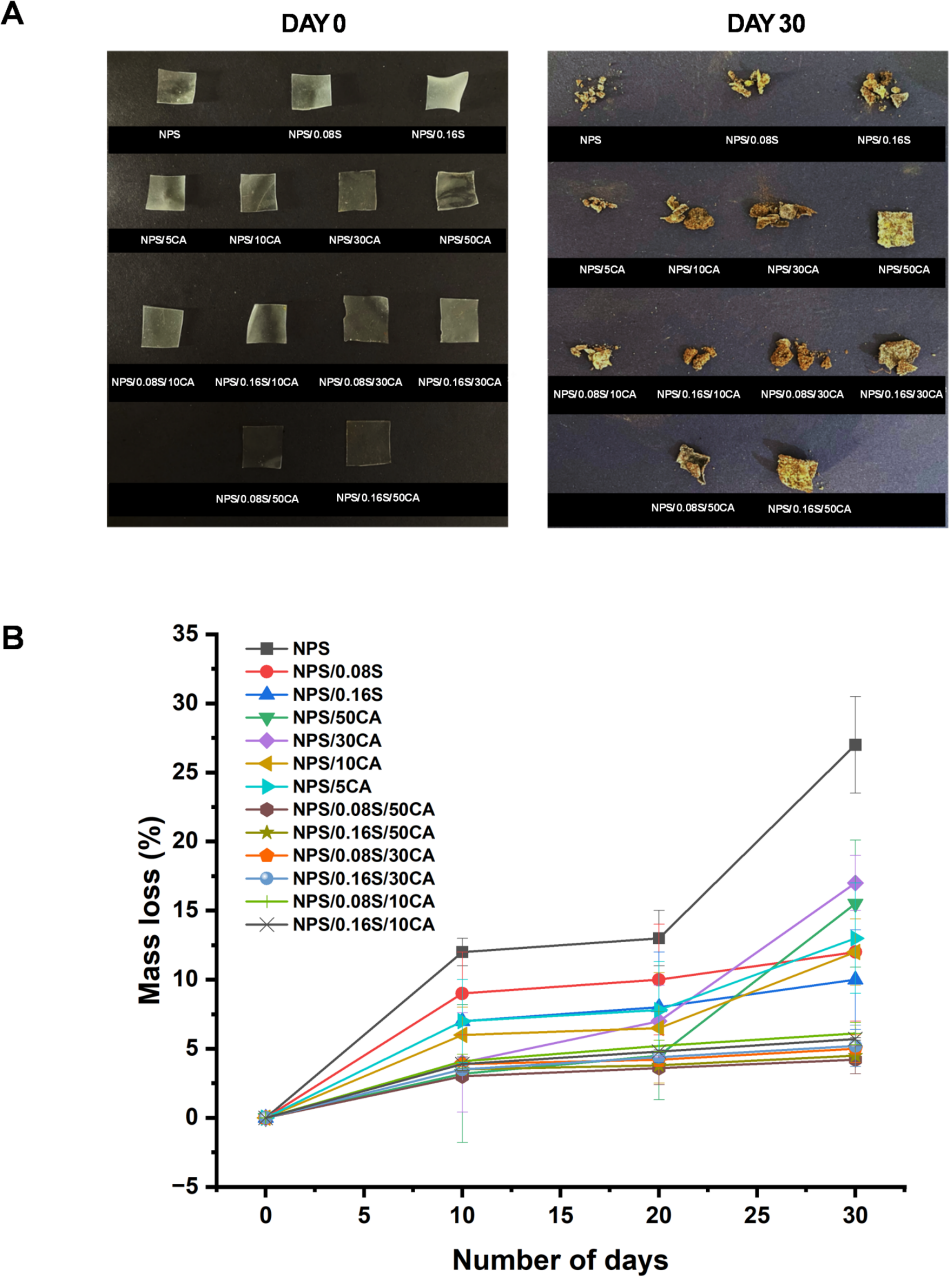
**(A)** Digital images showing degradation of synthesized elastomers in garden soil on day 0 and day 30; **(B)** Mass loss % curve exhibiting biodegradation of potato starch-based films in garden soil.

The soil burial test was conducted to evaluate the environmental sustainability of the starch-based films. While the primary application of these films is for cell culture, it is essential to assess their biodegradability to address broader environmental concerns related to biomedical waste disposal. This test provides insights into the degradation behavior of the films under natural conditions, which complements their potential application in eco-friendly biomedical solutions. As shown in Fig. 5A and B, the degradation profiles highlight the influence of citric acid and SiO_2_ on film longevity, offering valuable data for future applications requiring controlled degradation rates.

It was observed that the addition of SiO_2_had no appreciable impact. As a result of the antibacterial action of citric acid, which delays microbial growth and, in turn, slows down degradation, it was observed that films that had been crosslinked with citric acid degraded at slower rates which was in good agreement with previous studies^42^.

### Time dependent estimation of pH and absorbance properties of starch elastomers in DMEM

**Fig. 6:**
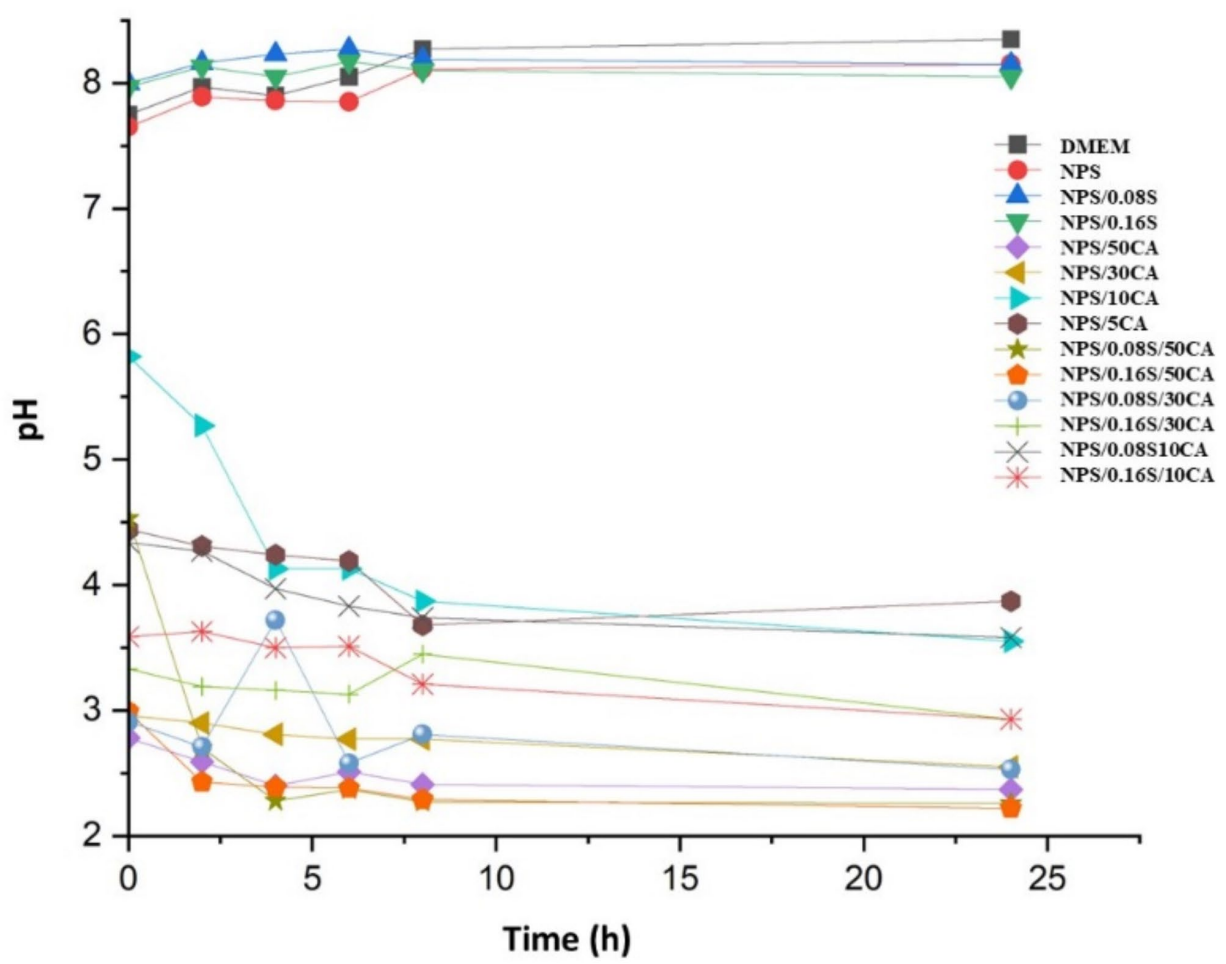
Change of pH of various starch-based films with DMEM at different intervals of time.

The pH measurements were conducted to evaluate the stability and suitability of the elastomeric starch-based films as a cell culture substrate (Fig. 6). Changes in pH can significantly impact cell behavior, including adhesion, proliferation, and viability. The release of acidic or basic byproducts from the films that could disrupt the culture environment ae monitored by evaluating the pH of the medium in contact with the films over time. This test is particularly relevant for applications in long-term cell culture, where maintaining a stable and physiologically relevant pH is crucial. The chosen time points provide a thorough view of the pH changes that took place for 24 h, enabling a thorough comprehension of the temporal dynamics of the films. The media’s pH was found to be more acidic for films that included 50% citric acid (CA). As a result, in further studies to lessen the overall acidity, films with a lower CA content (i.e., 10% w/w potato starch) were utilized.

**Fig. 7:**
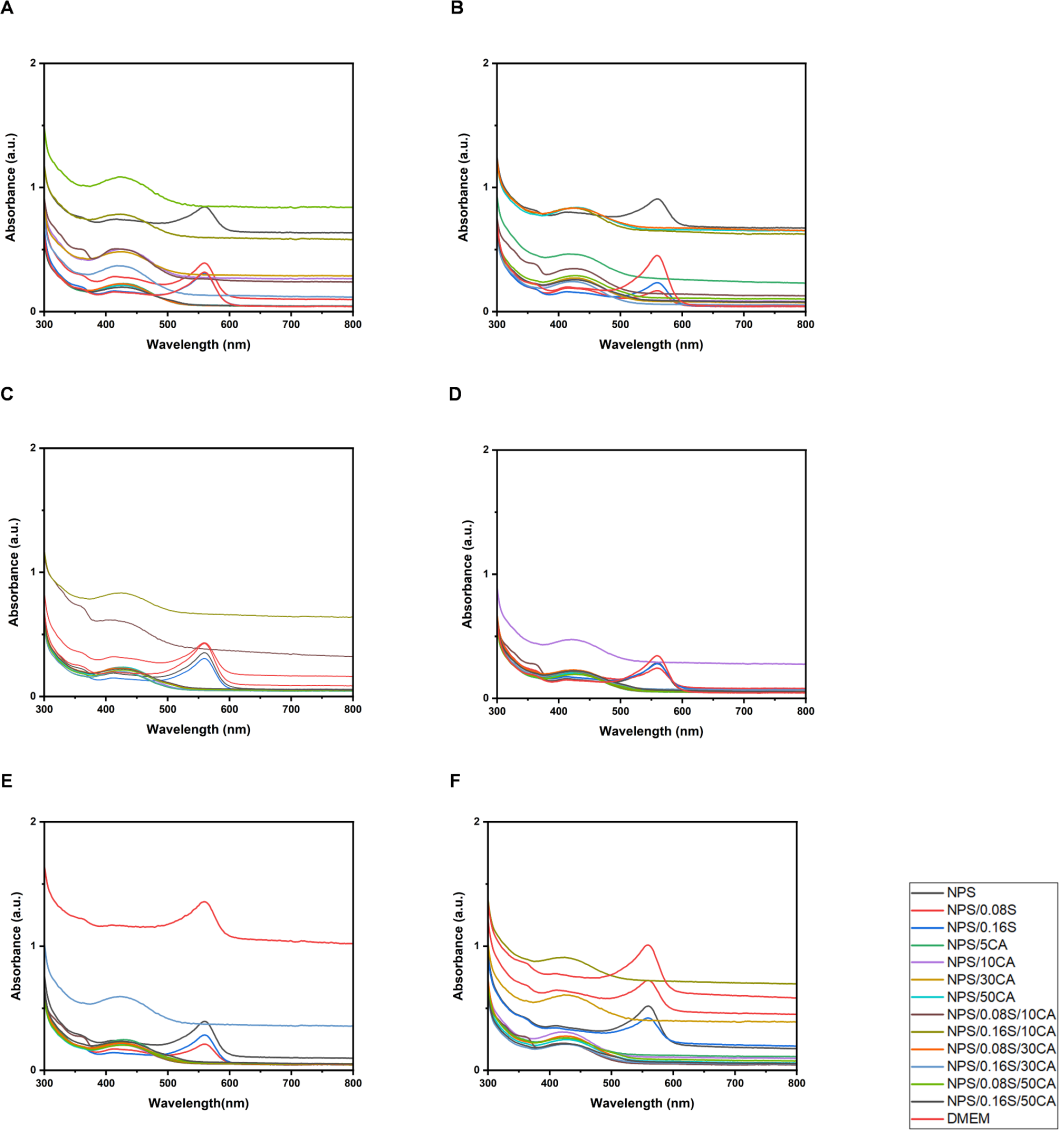
Absorbance spectrum of various potato starch-based elastomers with DMEM at different wavelengths at time intervals **(A)** 0 h, **(B)** 2 h, **(C)** 4 h, **(D)** 6 h, **(E)** 8 h, **(F)** 24 h.

The absorbance spectra (Fig. 7) suggests that both SiO_2_ and citric acid significantly influence the optical properties of the potato starch-based elastomers, particularly in their interaction with DMEM over time. The decrease in absorbance, especially in films containing SiO_2_ and CA, suggests possible degradation or leaching of these components. The differences in behavior among the films indicate that higher concentrations of these additives, i.e., SiO_2_ and CA, lead to greater initial interaction, which diminishes over time. A rapid decrease in absorbance could imply that these films interact heavily with the medium, potentially altering the medium’s composition, which could affect cell viability and proliferation. NPS/0.08 S/10CA shows a more moderate and consistent absorbance decrease, suggesting it might be a better balance between enhanced properties and stability in the medium.

### Biocompatibility

Biocompatibility is a key requirement for cell culture substrates, as it directly impacts cell adhesion, proliferation, and viability. Films that exhibit high biocompatibility provide a non-toxic environment for cells to grow and function optimally. Different cell lines constituting SiHa, HT-29 (cancerous) and HEK 293 (noncancerous) were seeded onto the bottom of 6 well plates coated with starch films. After 72 h, the films were removed from the culture medium and put into a new 6-well plate with a fresh culture medium. A control plate was also maintained without the elastomeric film. Very little cell confluency for all three cell lines was observed on the NPS and NPS/0.08 S films (Fig. S2 and 8). NPS/ 0.08 S/10CA showed better confluency than NPS and NPS/0.08 S films. However, the films constituting only NPS/10CA showed higher confluency. It was observed that even though cells maintained their original dimensions, they did not adhere to the film surface. Similar studies on biodegradable scaffolds for cell culture have reported enhanced biocompatibility when using organic crosslinkers like citric acid, further validating our findings^43^. A similar study investigated the biocompatibility of WPS-dicarboxylic acid derivatives synthesized from waste potato peels on NIH-3T3 and L929 murine fibroblast cells using MTT assays. It was found that the application of potato starch dicarboxylic acid derivatives did not result in significant apoptotic markers, such as reduced cell size or impaired intercellular contacts, indicating that the compounds were non-toxic^44^. The future of biodegradable starch polymers appears promising, particularly in sustainable industries and biomedicine. With continued research and development, these films could become a key player in reducing waste and providing eco-friendly, biodegradable alternatives for a variety of applications.

**Fig. 8:**
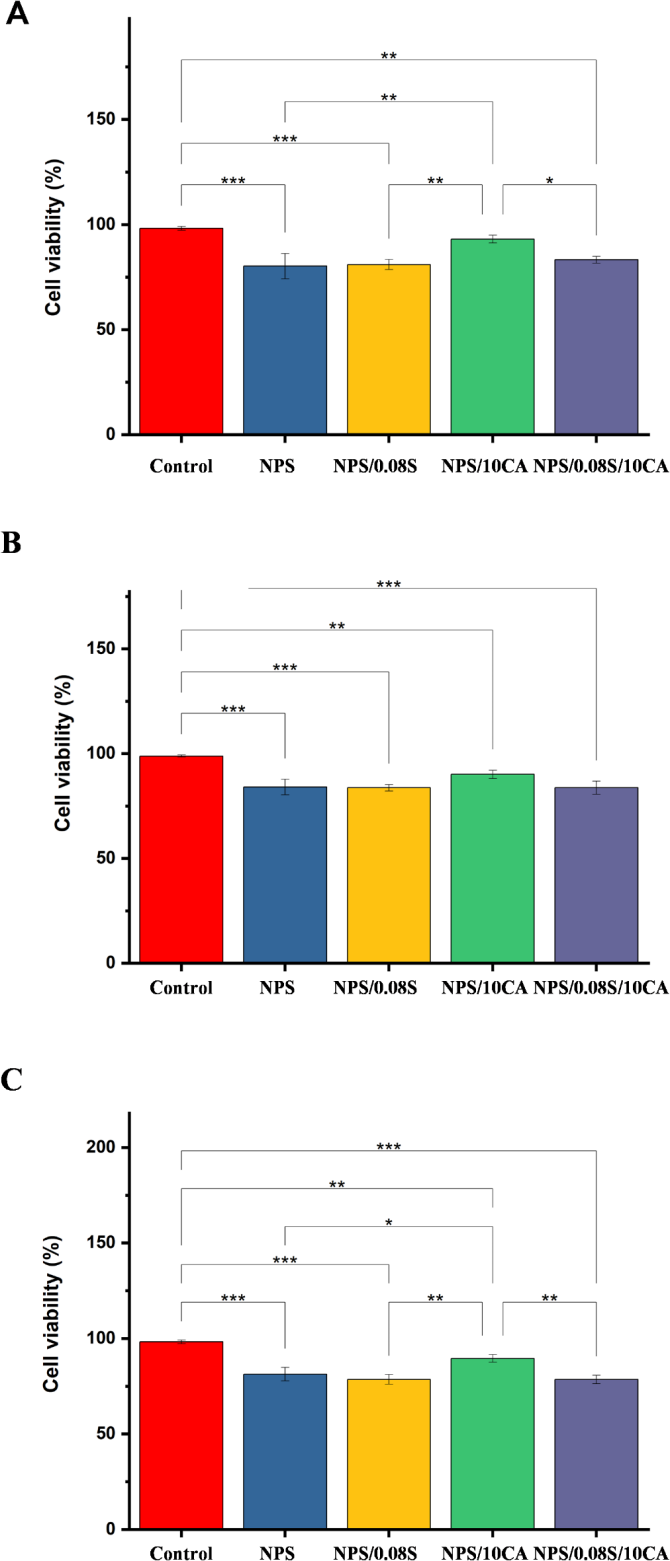
Graph showing cell viability (%) of **(A)** SiHa, **(B)** HT-29, and **(C)** HEK-293 cell lines in various starch-based elastomers.

## Conclusion

The incorporation of SiO₂ as a reinforcement filler and citric acid as a crosslinking agent in potato starch elastomers significantly enhanced their mechanical, barrier, and biocompatibility properties. Films containing SiO₂ demonstrated improved tensile strength, with a 20–25% increase compared to native starch films, while maintaining a reduction in elasticity of up to 15% at higher SiO₂ concentrations. Citric acid crosslinking further improved tensile strength by 25%, and combined SiO₂ and citric acid films exhibited a balanced performance with enhanced rigidity and flexibility.

Barrier properties such as WVTR and water solubility were also significantly improved. Citric acid-crosslinked films reduced WVTR by up to 30% compared to native films, while SiO₂ reduced solubility by approximately 15–20%. Films with both additives exhibited enhanced transparency in the visible spectrum (400–700 nm), achieving up to 90% transmittance, which is crucial for cell culture applications.

Biodegradation tests revealed that citric acid crosslinking delayed microbial growth, extending film longevity by 20% in soil burial conditions, while still being biodegradable. Biocompatibility assays with SiHa, HT-29, and HEK-293 cell lines demonstrated improved cell viability, with citric acid-containing films achieving up to 95% cell viability compared to 75% for native starch films.

These quantitative improvements highlight the potential of SiO₂ and citric acid-modified potato starch elastomers as sustainable, biodegradable, and biocompatible alternatives to conventional plastic substrates for biomedical applications.

**CRediT authorship contribution statement**.

**Pooja N**: data curation; investigation; visualization; methodology; writing – original draft. **Nafisa Yeshmin Ahmed**: data curation; investigation; visualization; methodology; writing – original draft. **Sib Sankar Mal**: supervision, writing – review & editing. **Guan-Yu Zhuo**: data curation; writing – review & editing. **Hemanth Noothalapati**: writing – review & editing. **Vishwanath Managuli**: methodology; writing – review & editing. **Bharath Prasad A S**: methodology; writing – review & editing. **Nirmal Mazumder**: conceptualization; supervision; review & editing.

## Electronic supplementary material

Below is the link to the electronic supplementary material.

Supplementary Material 1

## Data Availability

The authors declare that all the data supporting the findings of this study are available within the paper. Further data supporting the findings of this study are available from the corresponding author upon request.
