# Supplementary material for: Assessment of biocompatibility for citric acid crosslinked starch elastomeric films in cell culture applications
Source: Sci Rep. 2025 Feb 21;15:6427. doi: 10.1038/s41598-025-90933-0 (PMC11845471; doi:10.1038/s41598-025-90933-0)
Supplement: Supplementary file 1 — Supplementary Material 1 [file 41598_2025_90933_MOESM1_ESM.docx]

SUPPLEMENTARY DATA

Assessment of Biocompatibility for Citric Acid Crosslinked Starch Elastomeric Films in Cell Culture Applications

Pooja N^1#^, Nafisa Yeshmin Ahmed^1#^, Sib Sankar Mal^2^, Bharath Prasad A. S^3^, Guan-Yu Zhuo^4^, Hemanth Noothalapati^5,6,7^, Vishwanath Managuli^8^, Nirmal Mazumder^1,*^

*^1^Department of Biophysics, Manipal School of Life Sciences, Manipal Academy of Higher Education, Manipal-576104, Karnataka, India*

*^2^Department of Chemistry, National Institute of Technology, Suratkal-575025, Karnataka, India*

*^3^Department of Public Health Genomics, Manipal School of Life Sciences, Manipal Academy of Higher Education, Manipal-576104, Karnataka, India*

*^4^Institute of Biophotonics, National Yang Ming Chiao Tung University, Taipei 11221, Taiwan*

*^5^Department of Biomedical Engineering, Chennai Institute of Technology, Chennai-600069, Tamil Nadu, India*

*^6^Department of Chemical Engineering, Indian Institute of Technology Hyderabad, Kandi, Sangareddy-* *502285, Telangana, India*

*^7^Faculty of Life and Environmental Sciences, Shimane University, 1060 Nishikawatsu-Cho, Matsue 690-8504, Japan*

*^8^Department of Mechanical and Manufacturing Engineering, Manipal Institute of Technology, Manipal Academy of Higher Education, Manipal 576104, Karnataka, India*

#Equal Contribution

*Corresponding author: nirmal.mazumder@manipal.edu

Methods

Optical microscopy

The surface morphology of the starch films was analyzed using a bright-field microscope (Olympus BX51, Japan) at a magnification of 10x.

Results and discussion

Optical microscopy

Bright-field microscopic images at 10x magnification were used to depict the surface morphology of the synthesized films (Fig. S1). Potato starch elastomer showed an uneven surface when viewed under the microscope. Films with low concentrations of SiO_2_ showed a smooth surface and with higher concentrations, the films showed an increase in roughness. However citric acid crosslinked films showed a smoother than potato starch and SiO_2-_based films. Films with a combination of both citric acid and SiO_2_ showed relatively smooth surfaces.

Fig S1. Optical microscope images of potato starch elastomers: a. NPS, b. NPS/0.08S, c. NPS/0.16S, d. NPS/5CA, e. NPS/10CA, f. NPS/30CA, g. NPS/50CA, h. NPS/0.08S/10CA, i. NPS/0.16S/10CA, j. NPS/0.08S/30CA, k. NPS/0.16S/30CA, l. NPS/0.08S/50CA, and m. NPS/0.16S/50CA.


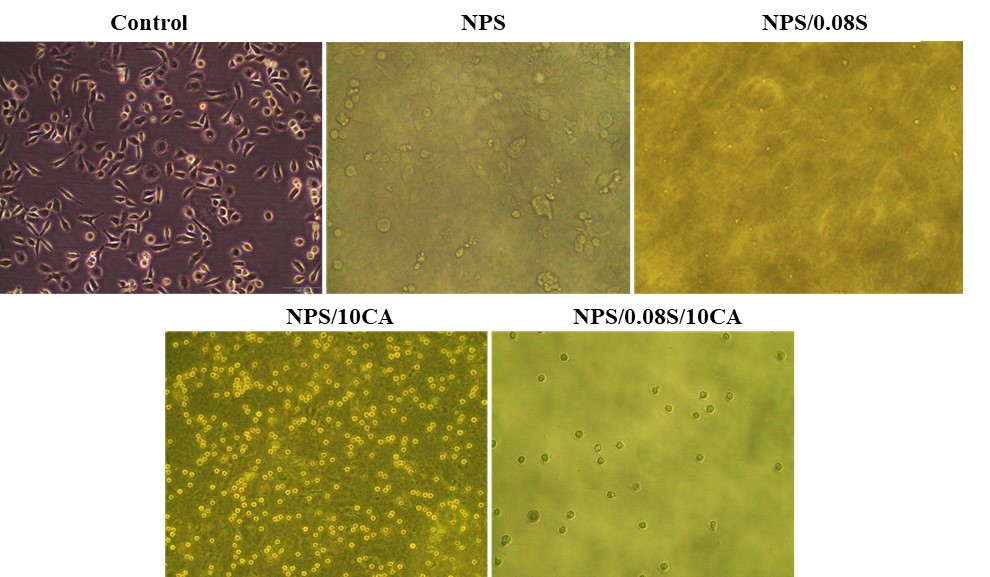
(A)


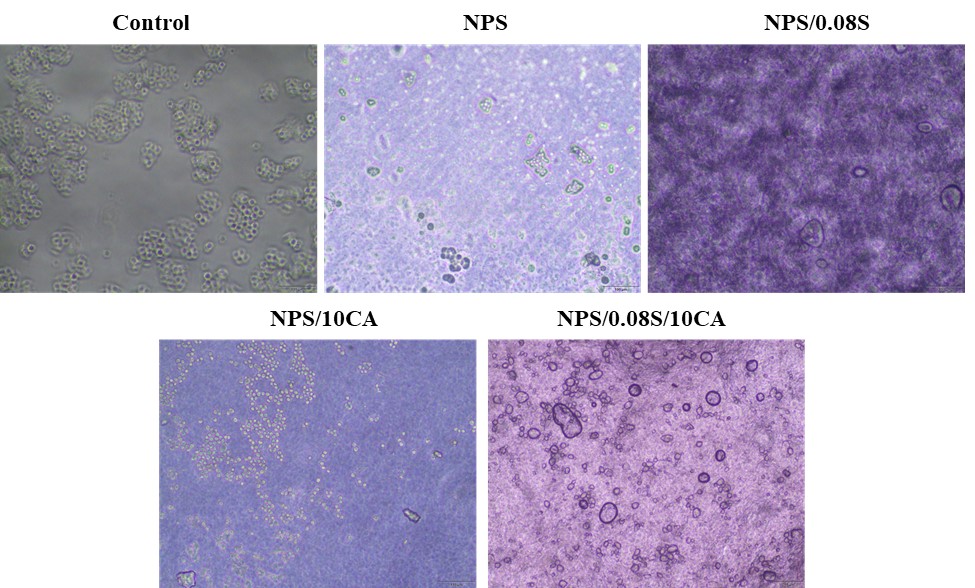
(B)


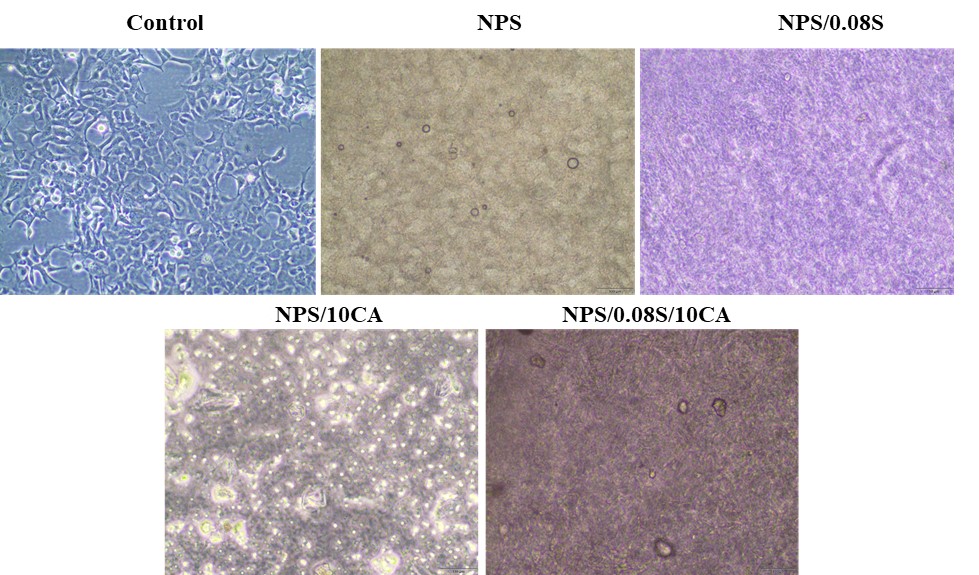
(C)

Fig. S2. Confluency of (A) SiHa, (B) HT-29, and (C) HEK-293 cell lines observed under the inverted microscope at 10x magnification after 72 h in a 6-well plate. PS: potato starch; SiO_2_: silicon dioxide; CA: citric acid.
